# Factors influencing full immunization coverage among 12–23 months of age children in Ethiopia: evidence from the national demographic and health survey in 2011

**DOI:** 10.1186/s12889-015-2078-6

**Published:** 2015-07-30

**Authors:** Yihunie Lakew, Alemayhu Bekele, Sibhatu Biadgilign

**Affiliations:** Ethiopian Public Health Association (EPHA), P.O Box 7117, Addis Ababa, Ethiopia; Ethiopian Public Health Association (EPHA), P.O Box 7117, Addis Ababa, Ethiopia; Independent Public Health Research Consultant, P.O Box 24414, Addis Ababa, Ethiopia

## Abstract

**Background:**

Immunization remains one of the most important public health interventions to reduce child morbidity and mortality. The 2011 national demographic and health survey (DHS) indicated low full immunization coverage among children aged 12–23 months in Ethiopia. Factors contributing to the low coverage of immunization have been poorly understood. The aim of this study was to identify factors associated with full immunization coverage among children aged 12–23 months in Ethiopia.

**Methods:**

This study used the 2011 Ethiopian demographic and health survey data. The survey was cross sectional by design and used a multistage cluster sampling procedure. A total of 1,927 mothers with children of 12–23 months of age were extracted from the children’s dataset. Mothers’ self-reported data and observations of vaccination cards were used to determine vaccine coverage. An adjusted odds ratio (AOR) with 95 % confidence intervals (CI) was used to outline the independent predictors.

**Results:**

The prevalence of fully immunized children was 24.3 %. Specific vaccination coverage for three doses of DPT, three doses of polio, measles and BCG were 36.5 %, 44.3 %, 55.7 % and 66.3 %, respectively. The multivariable analysis showed that sources of information from vaccination card [AOR 95 % CI; 7.7 (5.95-10.06)], received postnatal check-up within two months after birth [AOR 95 % CI; 1.8 (1.28-2.56)], women’s awareness of community conversation program [AOR 95 % CI; 1.9 (1.44-2.49)] and women in the rich wealth index [AOR 95 % CI; 1.4 (1.06-1.94)] were the predictors of full immunization coverage. Women from Afar [AOR 95 % CI; 0.07 (0.01-0.68)], Amhara [AOR 95 % CI; 0.33 (0.13-0.81)], Oromiya [AOR 95 % CI; 0.15 (0.06-0.37)], Somali [AOR 95 % CI; 0.15 (0.04-0.55)] and Southern Nation and Nationalities People administrative regions [AOR 95 % CI; 0.35 (0.14-0.87)] were less likely to fully vaccinate their children.

**Conclusion:**

The overall full immunization coverage in Ethiopia was considerably low as compared to the national target set (66 %). Health service use and access to information on maternal and child health were found to predict full immunization coverage. Appropriate strategies should be devised to enhance health information and accessibility for full immunization coverage by addressing the variations among regions.

## Background

Immunization is one of the key interventions to achieving the Millennium Development Goals (MDGs), especially the goal to reduce deaths among children under five years old [1]. Childhood vaccinations have been shown to be effective in protecting children against vaccine preventable diseases in low and middle income countries [2, 3]. Vaccines prevent more than 2.5 million child deaths per year [1].

The Expanded Programme on Immunization (EPI) was initiated by the World Health Organization (WHO) in 1974 to control vaccine preventable diseases worldwide [4]. In Ethiopia, EPI was first launched in 1980 and its vaccination schedule is based on the WHO recommendations [5, 6]. The national guideline was later published in 1988 and recently the Ethiopian immunization policy was updated in 2007 [7]. Tremendous progress in improving vaccination coverage has been made over the past four decades in many Africa countries [5]. Ethiopia has made encouraging improvements in accessibility and provision of primary healthcare services, especially since the establishment of the Health Extension Program in 2003. Recently, the country is also deploying and reinforcing the Women’s Development Army (WDA) initiative under the package of the health extension program. Immunization is a key component of the health extension program package. However, full vaccination coverage has not been completed in Ethiopia as planned. The full immunization coverage remained only 24.3 % in 2011 [8, 9] and as a result many children in Ethiopia have not received the benefits of full immunization [10]. The country has slightly higher infant and under 5 mortality rates that account for 59 and 88 deaths per 1000 live births, respectively [8] as compared to other developing countries [11].

Barriers for full vaccination coverage in other settings have been described and includes: lower parental education [12–14], lower income [13–15], being female gender of the child [16, 17], traditional and Muslim religions [18, 19], place of delivery and mothers received a postnatal check-up after 2 months of birth [20], household assets and expenditure, ethnicity, age and parity [21] were documented in Asian and African countries. In Ethiopia, malnutrition, vaccine preventable diseases and malaria were identified as the major causes of death among the under 5 children [22]. Furthermore, mothers’ knowledge about child immunization, postponing child immunization and perceived health institution support [23], institutional delivery and antenatal care (ANC) attendance [24], tetanus toxoid vaccine, place of residence and household visited by health workers [25], women’s decision making autonomy, number of under-five children in the household, mother’s education and proximity to health facilities [26] were factors identified through small scale studies in the country.

Though there have been few studies done fragmentally in different parts of Ethiopia, the social determinants of low vaccination coverage and non-uptake of immunization services have been poorly understood at the national level. Therefore, this study is intended to identify factors associated with full immunization coverage among children 12–23 months of age in Ethiopia.

## Methods

### Study setting

The 2011 Ethiopian Demographic and Health Survey (EDHS) was conducted in nine regional states of Ethiopia namely; Tigray, Afar, Amhara, Oromiya, Somali, Benishangul Gumuz, Southern Nations Nationalities and Peoples (SNNP), Gambella and Harari and two city Administrations (Addis Ababa and Dire Dawa). Ethiopia is one of the sub-Saharan countries found in the horn of Africa with a population of 73.5 million based on the 2007 national population and housing census [26].

### Sampling design

This study used the 2011 EDHS data. The survey employed a two-stage cluster sampling design. All women age 15–49 who were usual residents or who slept in the selected households the night before the survey were eligible. A total of 17,385 eligible women were identified and finally 16,515 women in the age group 15–49 were interviewed. The detailed methodology is found elsewhere [8]. Of all interviewed women, 11,654 gave birth in the past five years and 10,808 were interviewed for vaccination status. Children between 12–23 months of age are the youngest cohort who have reached the age by which they should be fully vaccinated. This age group was our target population for our analysis. So a total of 1,927 women with 12–23 months of children were extracted from the variable current age of child’s in the dataset. Information on a wide-range of potential independent variables (i.e. socio-demographic, economic, fertility history and health service use) were extracted accordingly. After reviewing the detailed data coding, further recoding of variables was done to better suit with other studies for comparison and intervention recommendations.

### Survey instrument and administration

The EDHS data include a women’s questionnaire that measures socio-demographic characteristics of the mothers, information on reproductive health and service use behaviors, as well as child-specific information for all births in the past five years from women of reproductive age group between 15–49 years. The 2011 EDHS collected information on vaccination coverage in two ways: (1) from vaccination cards shown to the interviewers and (2) from mothers’ verbal reports. If the cards were available, the interviewer copied the vaccination dates directly onto the questionnaire. When there was no vaccination card available for the child or if a vaccine had not been recorded on the card as being given, the respondent was asked to recall the vaccines given to her child.

### Measurement of variables

In this analysis, the dependent variable was full immunization coverage. According to the WHO guideline [27], “complete or full immunization” coverage is defined as a child has received a BCG vaccination against tuberculosis; three doses of DPT vaccine to prevent diphtheria, pertusis, and tetanus (DPT); at least three doses of polio vaccine; and one dose of measles vaccine. This dependent variable had five response categories: no, vaccination date on card, reported by mothers, vaccination marked on card and DK (don’t know). We recoded each variable into 0 and 1. No responses were recoded as “0” and labeled “not received the vaccine”, while the other responses “vaccination date on card, reported by mothers, vaccination marked on card” were recoded together as “1” and labeled “received the vaccine”. Then, we added all yes - zero scores and labeled them “Immunization status”. The immunization status was recoded as “0” if the child had received all the doses of vaccinations and categorized as “complete or full immunization” or “1” if the child had missed one or more doses of vaccinations and categorized as “incomplete immunization”.

The individual level exposure variables considered in this study were age of mothers, mother’s occupation, child death, parity, religion, women’s education, husband’s education, wealth index, birth order, awareness of community conversation (CC) program, sources of vaccination information, received postnatal check-up within 2 months after birth, antenatal care follow up of at least 4 times, place of delivery, number of living children in the household, sex of child and marital status. Whereas place of residence, agro-climatic zone and administrative regions were considered as the community level exposure variables.

### Definition of key terms

In this manuscript, ANC attendance refers to when women get services during pregnancy according to the WHO recommendations of at least four ANC visits for low-risk pregnant women and parity is defined as the number of children ever born. The wealth index constructed from household assets and characteristics available in the survey was used [8]. Occupational status was defined as non-paid and paid who were engaged in the areas of professional/technical/managerial, clerical, sales and services, skilled manual, unskilled manual and agricultural occupation classifications of the country.

### Statistical analysis

Descriptive statistics including prevalence and frequency distributions were used to determine the level of full immunization coverage by socio-demographic characteristics. Bivariate analysis was used to show the association between socio-demographic characteristics and full immunization coverage. Variables that were determined statistically significant at p-value <0.25 during bivariate analysis were considered for adjustment in the multivariable logistic regression model [28, 29]. This cut off point prevented removing variables that would potentially have an effect during multivariable analysis. A stepwise approach was used to assess the iteration of variables and to control potential confounders [30]. In the multivariable model, odds ratio with 95 % CI was used. A multi-collinearity test was done and as a result marital status and birth order were omitted from the multivariable analysis because of collinearity with variance inflation factors (VIF) of greater than 10 [31]. Sample weights were applied in order to compensate for the unequal probability of selection between the strata that has been geographically defined as well as for non-responses. A detailed explanation of the weighting procedure can be found in the DHS 2011 [8]. The “svy” command in STATA version 11 (Stata Corporation, College Station, TX, USA) was used to weight the survey data.

### Ethical clearance

The 2011 EDHS data is available to the general public by request in different formats from the Measure DHS website [http://www.measuredhs.com]. We submitted a request to the Measure DHS by briefly stating the objectives of this analysis and thereafter received permission to download the children’s’ dataset in SPSS format.

## Results

A total of 1,927 women with children between 12–23 months of age were included in the analysis. As indicated in Table 1, the overall prevalence of fully immunized children in Ethiopia was 24.3 %. Specific vaccination coverage for DPT3, Polio3, Measles and BCG were 36.5 %, 44.3 %, 55.7 % and 66.3 %, respectively. About 29 % of mothers who had vaccination cards had fully immunized their children of age 12–23 months. The coverage in urban and rural settings was 48.2 % and 20.4 %, respectively.

**Table 1:** Prevalence of full vaccination coverage among women with children of age 12–23 months in Ethiopia, 2011

Variables	Weighted total number of respondents	Percent of full vaccination with 95 % CI
Regions		
Tigray	129	58.9 (50.27-67.16)
Afar	18	8.6 (1.91-32.11)
Amhara	446	26.3 (22.31-30.47)
Oromiya	811	15.6 (13.28-18.28)
Somali	51	16.6 (7.56-27.62)
Benishangul-Gumuz	23	23.6 (8.43-41.80)
SNNP	391	24.1 (20.00-28.47)
Gambella	8	15.5 (0.63-48.03)
Harari	5	34.1 (7.35-81.76)
Addis Ababa	43	78.7 (65.05-89.28)
Dire Dawa	7	58.6 (21.60-87.73)
Residence		
Urban	274	48.2 (42.29-54.10)
Rural	1,656	20.4 (18.52-22.40)
Agro-climatic zone		
Lowland	308	21.3 (17.12-26.28)
Highland	1,622	24.9 (22.85-27.06)
Age		
15-24	518	24.4 (20.77-28.16)
25-34	1035	24.0 (21.43-26.63)
35-49	378	25.1 (20.95-29.69)
Wealth index		
Poor	860	17.5 (15.12-20.21)
Middle	394	18.2 (14.69-22.32)
Rich	675	36.6 (33.02-40.28)
Maternal Education		
No formal education	1,307	20.1 (18.02-22.36)
Primary	522	28.3 (24.61-32.34)
Secondary	59	57.0 (44.80-69.72)
Higher	43	57.7 (43.10-72.12)
Religion		
Orthodox	750	32.9 (29.64-36.36)
Catholic	18	28.1 (10.96-51.27)
Protestant	457	21.1 (17.46-24.92)
Muslim	660	16.6 (13.97-19.66)
Traditional and others	45	25.4 (13.58-38.51)
Maternal occupation		
Not paid	894	22.3 (19.62-25.08)
Paid	1,036	26.0 (23.36-28.70)
Child Death		
Has not death	1,340	24.7 (22.45-27.06)
Has death	589	23.3 (19.98-26.80)
Parity		
1	346	29.1 (24.58-34.15)
2-5	1,080	24.8 (22.31.27.46)
6+	504	20.0 (16.72-23.71)
Husband education		
No formal education	937	21.0 (18.51-23.72)
Primary	796	23.1 (20.29-26.14)
Secondary and higher	105	39.2 (30.07-48.62)
Birth order		
1	358	29.8 (25.31-34.79)
2-5	1,075	24.3 (21.79-26.91)
6+	497	20.3 (16.96-24.03)
Awareness of community conversation program		
No	297	36.6 (31.36-42.30)
Yes	220	38.4 (31.94-44.74)
ANC attendance		
No	1,507	20.4 (18.39-22.46)
Yes	349	41.7 (36.74-47.07)
Place of delivery		
Home	1,694	20.4 (18.56-22.40)
Health facility	236	52.7 (46.16-58.86)
Source of information		
Mothers’ self-report	1376	11.2 (9.16-12.94)
Card/document	554	57.0 (52.89-61.12)
Marital status		
Never married	11	61.3 (33.64-87.22)
Currently married	1,796	23.8 (21.85-25.79)
Formerly married	122	27.7 (20.46-36.32)
Sex of child		
Male	1,010	23.1 (20.55-25.74)
Female	920	25.7 (22.91-28.55)
Mothers received a postnatal check-up within 2 months after birth		
No	1,663	21.6 (76.39-80.34)
Yes	190	48.6 (72.15-83.84)
Number of living children in the household		
1	374	30.1 (25.49-34.74)
2+	1556	22.9 (20.84-25.02)

Children whose mother’s educational level higher and secondary had full immunization coverage of 57.7 % and 57 %, respectively. Children whose families’ wealth index grouped as poor, middle and rich categories had immunization coverage of 17.5 %, 18.2 % and 36.6 %, respectively. The full immunization status among children whose mothers age 15–24, 25–34 and 35–49 was 24.4 %, 24 % and 25.1 %, respectively. Factors where immunization coverage was higher included: in Addis Ababa, Tigray and Dire Dawa administrative regions, mothers aware of CC program, never married women, female children, highlanders, Orthodox followers, single parity and first birth order, husbands’ secondary education, born at health facility, mothers attended ANC and mothers who have a postnatal check-up within 2 months after birth had full vaccination coverage in the country (Table 1).

The multivariable analysis in Table 2 showed that full immunization coverage was highly associated with sources of information from the vaccination card. Children whose mothers showed the vaccination card during interview were 7.7 times [AOR with 95 % CI; 7.7 (5.95-10.06)] more likely to receive full vaccination than those whose mothers verbally reported. Mothers who received postnatal check-up within 2 months after birth from health facilities were 1.8 times [AOR 95 % CI; 1.8 (1.28-2.56)] more likely to receive full vaccination than those who did not check after delivery. Children born to mothers who were aware of the CC program were 1.9 times [AOR 95 % CI; 1.9 (1.44-2.49)] more likely to get full vaccination than their counterparts. There was a 40 % [AOR 95 % CI; 1.4 (1.06-1.94)] more likely in receiving full vaccination among children born to mothers of rich wealth index group compared with children from women of poor wealth index group. Women from Afar [AOR 95 % CI; 0.07 (0.01-0.68)], Amhara [AOR 95 % CI; 0.33 (0.13-0.81)], Oromiya [AOR 95 % CI; 0.15 (0.06-0.37)], Somali [AOR 95 % CI; 0.15 (0.04-0.55)] and Southern Nation and Nationalities People [AOR 95 % CI; 0.35 (0.14-0.87)] administrative regions were less likely to fully vaccinate their children compared to women who reside in Addis Ababa [Table 2]. After adjusting for all variables, antenatal care (ANC) attendance, place of delivery, religion, birth order, number of living children in the household, sex of child, place of residence, agro-climatic zone, maternal education, husbands’ education, age, parity, child death, and maternal occupation were no longer associated with full immunization in the multivariable analysis.

**Table 2:** Multivariable logistic regression to identify factors associated with full immunization coverage among women with children 12–23 months of age in Ethiopia, 2011

Variables	Crude OR with 95 % CI	Adj OR with 95 % CI
Wealth index		
Poor	1.00	1.00
Middle	1.0 (0.77-1.43)	0.85 (0.59-1.22)
Rich	2.7 (2.1-3.44)***	1.4 (1.06-1.94)*
Region		
Addis Ababa	1.00	1.00
Tigray	0.39 (0.17-0.87)*	0.77 (0.29-2.02)
Afar	0.03 (0.01-0.16)***	0.07 (0.01-0.68)*
Amhara	0.10 (0.04-0.21)***	0.33 (0.13-0.81)*
Oromiya	0.05 (0.02-0.11)***	0.15 (0.06-0.37)***
Somali	0.05 (0.02-0.15)***	0.15 (0.04-0.55)**
Beneshangul Gumuz	0.08 (0.02-0.28)***	0.27 (0.07-1.13)
SNNP	0.09 (0.04-0.19)***	0.35 (0.14-0.87)*
Gambella	0.05 (0.01-0.39)**	0.16 (0.11-1.60)
Harari	0.14 (0.02-1.01)	0.30 (0.03-3.32)
Dire Dawa	0.38 (0.07-2.09)	0.90 (0.12-6.92)
Received check-up within 2 months of birth		
No	1.00	1.00
Yes	3.4 (2.52-4.65)***	1.8 (1.28-2.56)*
Awareness of CC program		
No	1.00	1.00
Yes	2.5 (1.98-3.08)***	1.9 (1.44-2.49)***
Sources of information		
Mothers’ self-report	1.00	1.00
Card (document)	10.5 (8.31-13.37)***	7.7 (5.95-10.06)***

(***)P < 0.0001, (**)P < 0.001), (*)p < 0.05

## Discussion

This study identifies factors associated with full immunization coverage of children 12–23 months of age in Ethiopia. The percentage of children who are fully immunized remains far below the goal of 66 % coverage set in the country’s health sector development plan (HSDP) IV [22]. The national prevalence of fully immunized children was lower as compared to some district level survey findings such as 36 % in West Shewa [32], 41.7 % in Southern Ethiopia [23], 37 % in southwestern Ethiopia [26] and 51 % in northern Ethiopia [33]. The difference could be explained by the fact that the health and demographic survey was conducted in a broader population which is an aggregate of very remote and urbanized areas in the country.

A national EPI coverage survey conducted in Ethiopia conducted showed that full immunization coverage in 2006 was 49.9 % [34] which is higher than the 2011 EDHS findings. Full vaccination coverage in Ethiopia is lower compared to other sub-Sahara African countries like Kenya 57.7 % [35], Malawi 51 % [36], Uganda 68 % [37] and other countries like India (39 %) [38], Brazil 47 % [39] and Pakistan 71.9 % [40]. The differences in the coverage rates could be explained by differences in the methods to generate data, scope of the surveys and differences in the health service coverage’s including immunization service among these countries. The EDHS uses relatively strict indices of measurements for calculating full immunization coverage, including a composite score of BCG, measles, DPT (1, 2, 3) and Polio (1, 2, 3). Furthermore, the health and demographic survey was conducted in a broader population and reported as an aggregate of very remote and urbanized areas in the country, which can explain why the findings differ from local studies. The findings of the specific vaccines’ coverage reported in this paper is higher than that of the study conducted in France among the Haji pilgrims (i.e. the vaccination rates for tetanus (18.9 %), diphtheria (14.7 %) and poliomyelitis (15.0 %) [41]. This might be explained by differences in the uptake of immunization services due to cultural variations among the two populations. Furthermore, there might be large dropouts of completing subsequent doses of a specific vaccine particularly for DPT and Polio. This can be further exemplified in this analysis that DPT and Polio had only 36.5 % and 44.4 % coverage compared to BCG and measles immunization coverage which had 66 % and 55 % coverage, respectively.

In order to identify factors associated with full immunization coverage in the country, a range of social factors available in the EDHS data were selected based on literature review. Of the selected variables and included in the analysis: wealth index, received postnatal check-up within 2 months after birth, sources of information for vaccination status, administrative regions and mother’s awareness of CC program were significantly associated with full immunization coverage.

In this study, rich families were more likely to fully vaccinate their children compared to poor families, which is consistent with a study findings in Bangladesh [42] and southwestern Ethiopia [23]. In our analysis, utilization of postnatal care service is found to be associated with completion of child immunization. Children who received check-up within two months after birth were more likely to be fully vaccinated which is consistent with a study in southwest Ethiopia [23]. Similarly, an increased likelihood of full vaccination status among children whose mothers received a postnatal check-up within 2 months after birth was found in Tanzania [20]. In contrast, a study in Burundi also revealed that children were less likely to have full vaccination coverage among mothers who received a postnatal check-up within 2 months after birth. What so ever result is documented, a frequent contact with the health care system either for antenatal or postnatal care would increase the possibility to getting fully immunized children even though there are many factors influencing to do so in developing countries.

Awareness of the CC program increases the knowledge of mothers to deliver in health facilities then potentially leads to vaccine their children. In this study, mothers’ awareness of the CC program has a positive effect for full vaccination coverage. This might be due to the fact that mothers have received health education on the importance of immunization through CC programme. The CC programme is one of the strategies for social mobilization through creating a dialogue among a community members targeting at raising awareness and behavioral change. It is being implemented by the Ethiopian Ministry of Health and the HIV/AIDS Prevention and Control Office (HAPCO) with development partners. It was primarily designed for HIV/AIDS control and prevention but currently it has been used for other health issues including child immunization that is being addressed through CC to obtain behavioral changes. A Bangladesh study showed that mothers with mass media exposure (regularly watching TV and listening to radio) were more likely to fully immunize their children than mothers who had no mass media exposure [42]. A study carried out in rural Nigeria demonstrated that mothers with a higher knowledge score were more likely to fully immunize their children [43]. So creating awareness inducing approach through novel CC model is vital to mitigate the knowledge deficit that has profound effect to the community to increase the awareness on usage of the health facility and immunization services.

In this analysis, significant variation in vaccination coverage among administrative regions of the country was observed. This can be explained in other studies as differential access to vaccinations due to either transportation issues [12, 44] or difficulties in supply chain coverage or presence of demand related barriers [45]. Factors including staffing shortages and commitment, management of health facilities, quality of care available, and community communication can also account for differences in quality of service delivery and therefore may account for differences in vaccination coverage [46–48]. Improving vaccination access across all regions of a country could have a multifaceted effect on healthcare delivery by increasing health worker productivity and coverage area as well as maximizing the efficiency of their health interventions [44].

This study has its own limitations. The actual proportion of children who have a vaccination card may be higher because in some areas the cards are lost. Furthermore, information obtained from mothers or care givers on the vaccination status of their children is not as reliable as that of vaccination card due to recall bias. This study did not also address the supply side factors including access and availability of services for full vaccination coverage. In particular, the study lacks the effect of the vaccine management system and service delivery related factors like logistics, access to health care and trained human resources as predictors of child immunization coverage. This study did not cover the infrastructure and human resources that might have contributed for low child immunization coverage. Despite these limitations, the present study attempted to assess many predictors for full child immunization coverage using a nationwide representative survey data of the 2011 EDHS that can inform policy and program actions.

## Conclusion

The overall full immunization coverage of the country was considerably low compared to other studies within the country and abroad. Variations in the prevalence of immunization coverage across administrative regions were observed. The urban settings have higher immunization coverage compared to the rural settings. Independent predictors of full immunization coverage were sources of information for vaccination status, received postnatal check-up within two months after birth, awareness of CC, rich wealth index and administrative regions. The Federal Ministry of Health and development partners should exert the utmost concerted efforts at all level to increase full immunization coverage targeting their activities to those hard to reach and developing regions of the country. Regional based vaccine implementation program actions to enhance vaccine coverage among administrative regions of the country are warranted. Deploying and reinforcing the Women’s Development Army (WDA) initiative under the package of the health extension program could able to enhance the mothers’ communication around their health issues including immunization to improve full vaccination coverage. Continuous awareness raising interventions is vital through CC on the use of immunization at the grass root level by health extension workers and other development agents. This helps to sustainably improve the awareness, knowledge of the mothers and the general community on the benefits of immunizing their children. This ultimately increases the uptake of vaccination in the community. A community as well as facility based studies are commendable to capture other important contextual factors which is not addressed in this study.
